# Application of Cu(I)-catalyzed azide–alkyne cycloaddition for the design and synthesis of sequence specific probes targeting double-stranded DNA

**DOI:** 10.3762/bjoc.12.128

**Published:** 2016-06-30

**Authors:** Svetlana V Vasilyeva, Vyacheslav V Filichev, Alexandre S Boutorine

**Affiliations:** 1 Institute of Chemical Biology & Fundamental Medicine, SB of RAS, pr. Lavrent’eva 8, 630090 Novosibirsk, Russia; 2 Institute of Fundamental Sciences, Massey University, Private Bag 11-222, 4442 Palmerston North, New Zealand; 3 Structure and Instability of Genomes, Sorbonne Universités, Muséum National d'Histoire Naturelle, INSERM U 1154, CNRS UMR 7196, 57 rue Cuvier, C.P. 26, 75231 Paris cedex 05, France

**Keywords:** binding affinity, click chemistry, Cu(I)-catalyzed azide–alkyne cycloaddition, pyrrole–imidazole polyamides, sequence specificity: DNA, triplex-forming oligonucleotides

## Abstract

Efficient protocols based on Cu(I)-catalyzed azide–alkyne cycloaddition were developed for the synthesis of conjugates of pyrrole–imidazole polyamide minor groove binders (MGB) with fluorophores and with triplex-forming oligonucleotides (TFOs). Diverse bifunctional linkers were synthesized and used for the insertion of terminal azides or alkynes into TFOs and MGBs. The formation of stable triple helices by TFO-MGB conjugates was evaluated by gel-shift experiments. The presence of MGB in these conjugates did not affect the binding parameters (affinity and triplex stability) of the parent TFOs.

## Introduction

**Figure 1:**
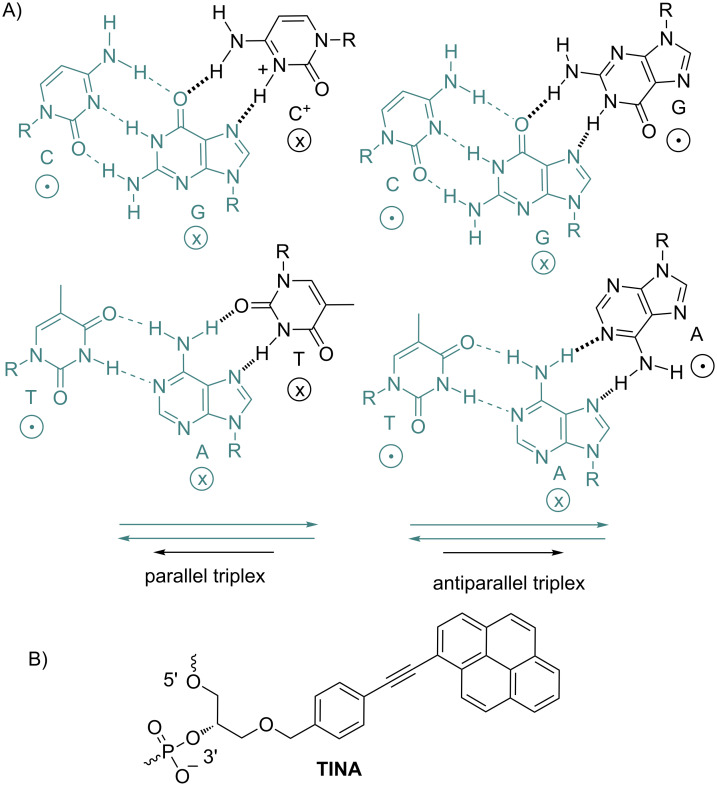
A) Formation of nucleotide triplets in parallel and antiparallel (relatively to polypurine strand) complexes. The relative orientations of DNA backbones are shown by the symbols “
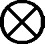
” and “
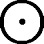
”. The duplex is in green, TFO is in black. B) Chemical structure of the TINA molecule inserted in the TFO structure of the DNA triplex as a bulge.

The recognition and detection of specific sequences in native genomic double-stranded DNA (dsDNA) is of significant importance for the development of efficient gene therapies and in vivo gene labeling [1–3]. Besides natural and engineered peptides or proteins, two synthetic substances are known to recognize and bind dsDNA in a sequence-specific manner: triplex-forming oligonucleotides (TFOs) [4–5] and pyrrole–imidazole polyamide minor groove binders (MGBs) [6–7]. TFOs recognize polypurine stretches in genomic DNA and bind in the major groove of dsDNA. Cytosine-containing-TFOs (CT-TFOs) form parallel triplexes with dsDNA (Figure 1) but these complexes are not stable at neutral pH: cytosine in the TFOs has to be protonated in order to form a hydrogen bond with guanosine of the duplex. In contrast, G-rich TFOs that can bind to dsDNA in a parallel and antiparallel fashion are insensitive to pH, but the formation of exceptionally stable internal structures (G-quadruplexes) can inhibit the triplex assembly. The exploration of the triplex technology by the scientific community would be possible if TFOs can be efficiently delivered to the nucleus and bind to dsDNA with high affinity and sequence-specificity.

MGBs recognize target sequences according to the well-established rules [6] with high affinity and without pH or ionic strength limitations. They penetrate into living cells and reach genomic DNA, so they were successfully used for both gene expression regulation and the synthesis of fluorescent probes for DNA imaging in fixed and live cells [1–3,8–9]. However, the length of the sequence recognized by the classical hairpin polyamides (5–6 base pairs) is much shorter than that for TFOs (>10–12 base pairs) that results in promiscuous binding events.

To resolve the mentioned problems, a chemical conjugation of several DNA-binding ligands (TFOs, MGBs) was used in order to profit from useful properties of both components in the resulting TFO-MGB conjugates [10–11] and MGB tandems [12–15]. Recently a new class of TFOs containing covalently linked intercalating pyrene moieties, so-called "twisted intercalating nucleic acids" (TINA), has been described [16–17]. TINA-TFOs form stable and specific triplexes with target dsDNA at physiological conditions. It has been shown that the most stable complexes are formed by antiparallel G-rich TINA-TFOs [17–18] (Figure 1).

The conjugation of TINA-TFOs to MGBs should allow for recognition of longer sequences in dsDNA than for the parent components thus providing improved affinity and sequence specificity [11]. Using carbodiimide activation or Mukayama reagents [19–21], TFO-MGB conjugates have been synthesized [11,22–24]. However, this method is not universal because it requires solubility of all reagents in organic solvents, which is incompatible with in vivo and in situ applications. In addition, according to our experience, these reactions are not suitable for TINA-TFO derivatives due to excessive formation of side products.

**Figure 2:**
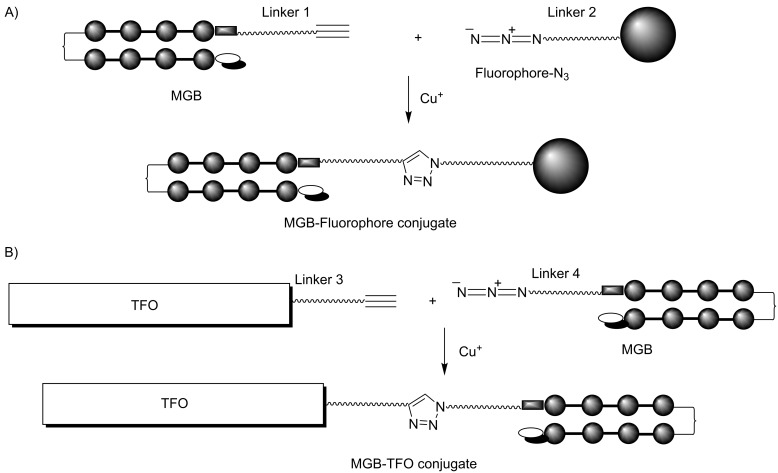
Synthesis of MGB-fluorophore (A) and MGB-TFO (B) conjugates using CuACC. Linker length and composition can be varied.

Copper(I)-catalyzed azide–alkyne cycloaddition (CuAAC) as a variation of the Huisgen 1,3-dipolar cycloaddition has become a widely used conjugation method in which the stereoselective formation of 1,2,3-triazoles can connect several components in one molecule [25–26]. CuAAC belongs to the class of chemical processes called “click-chemistry” and it is also a biorthogonal reaction because functional groups of natural biopolymers are not affected and do not participate in chemical transformations. So, this reaction is suitable for the synthesis of different biologically important conjugates including polyamide-TFO conjugates and fluorescent probes based on these components. Several methods avoiding the presence of cytotoxic copper ions have been also described [27–29]. Baskin et al. demonstrated the fluorescent labeling of proteins directly in living cells by copper-free "click chemistry" [29]. Alkyne or azide groups can be inserted into both TFO and MGB using enzymatic or chemical methods during matrix or solid-phase synthesis [2,30–31] or post-synthetically using described conjugation methods [32]. In order to achieve a perfect fit between MGB-conjugates and dsDNA target sequences, the length and the flexibility of the linker that connects two components (polyamide and TFO or fluorophore) in the conjugates should be optimized. It becomes vital for TFO-MGB conjugates because individual components, TFO and MGBs, bind in the major and minor groove of dsDNA, respectively. The linker length optimization can be achieved by combination of "classic" methods of conjugation with "click chemistry". Preliminary results of this work were presented as a poster paper at a conference [33]. In the first part of the present article, we describe the synthesis of various linkers bearing different functional groups for simple and accessible introduction of azide and alkyne groups into biopolymers, fluorophores and ligands. Then, these linkers were introduced into the structure of MGBs and TFOs and used for the synthesis of MGB-fluorophore and MGB-TFO conjugates via CuAAC (Figure 2). Properties of the conjugates obtained are discussed.

## Results and Discussion

### Synthesis of bifunctional linkers

**Figure 3:**
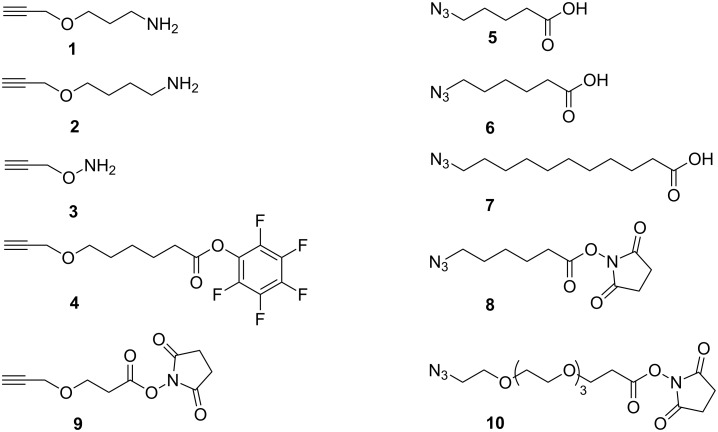
Bifunctional linkers for conjugation of oligonucleotides and polyamides using CuACC.

Two components in MGB-TFO conjugates must be connected by a linker, which is at least 12 chemical bonds long [10–11,34]. Several different bifunctional linkers were designed and synthesized (Figure 3). All of them contain, on one side, an alkyne or azide group for "click chemistry” and, on the other side, either an amino group for interaction with electrophiles (for example, activated terminal phosphates of oligonucleotides) or an activated carboxylic ester for reactions with nucleophiles (for example, terminal amino groups of MGBs). The linkers have various lengths and chemical natures and can be coupled either directly during a solid-phase synthesis or post-synthetically to pre-synthesized or commercially purchased ligands.

The first three linkers (**1**–**3**) are designed for functionalization of commercial phosphorylated TFOs according to established protocols [32]. They contain an amino group for coupling to an activated terminal phosphate of oligonucleotides and an alkyne group for CuAAC reactions. The linker **4** is designed for functionalization of either commercial amino-modified oligonucleotides or amine-bearing polyamides. It contains an alkyne group for CuAAC and an activated ester for the acylation of amino groups. The linkers **5**–**7** of different lengths contain an azide group for CuAAC and a carboxyl group that can react after activation with the terminal amino groups of polyamides or of amino-modified oligonucleotides (see Supporting Information File 1 for full experimental data and physicochemical characteristics of compounds). In addition, commercially available reagents **9**, with an oxygen atom in the chain, and **10** possessing several ethylene glycol moieties were used to increase the solubility of modified polyamides in water.

### Selection of the target DNA and design of TFOs and MGBs

**Figure 4:**
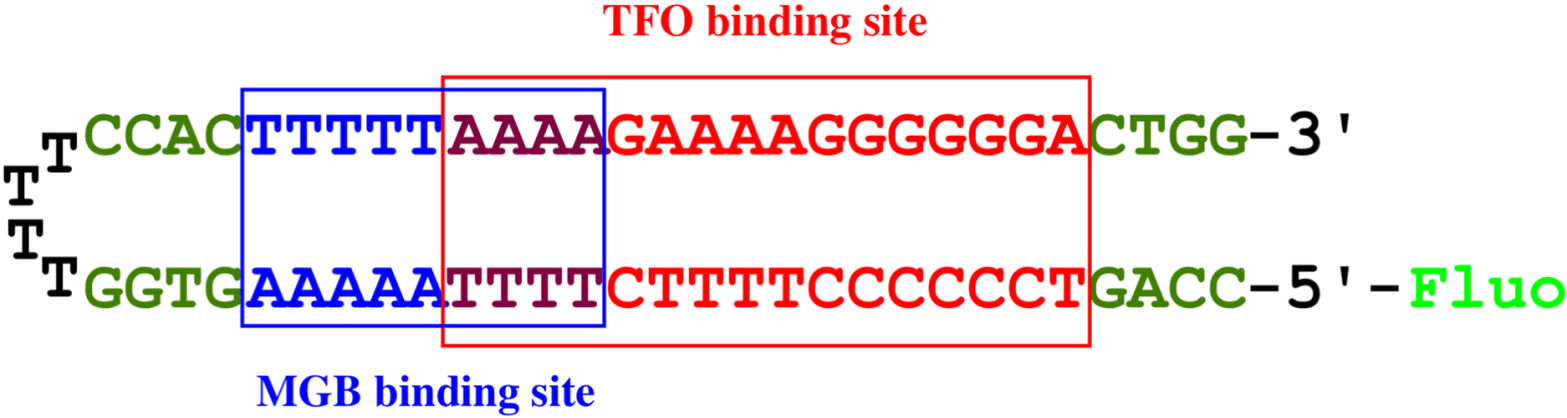
The target duplex contains a 29 base pair fragment from HIV proviral DNA [35] and a T4 hairpin is connecting two strands: a 16 base pair polypurine-polypyrimidine tract (TFO-binding site – is indicated by a red rectangle), an adjacent overlapping poly(dA:dT) tract (MGB-binding site – by a blue rectangle). Fluo is a fluorescein label.

The polypurine tract of the HIV-1 provirus is a valuable target for MGB-TFO conjugates [35]. It has a 16-bp polypurine site suitable for DNA triplex formation and overlaps with an A:T tract which is an ideal target for easily synthesized MGB containing only *N*-methylpyrrole moieties (Figure 4). For convenience of our work, a fluorescently labeled T4 hairpin covalent duplex containing the 29 base pair HIV polypurine sequence was used.

Thus, two sequences for the design of TFOs were selected. The first sequence is 5′-TTTTCTTTTCCCCCCT that forms parallel triplexes, and the second one is 5′-AGGGGGGTTTTGTTTT that forms antiparallel triplexes on the target dsDNA. The last sequence has six consecutive guanosines that promote the formation of a G-quadruplex that hinders DNA triplex formation. In order to destabilize the G-quadruplex and to form the DNA triplexes, TINA modification can be introduced in the structure of TFO (TINA-TFO) [17].

A simple hairpin pyrrole hexamer γPyPyPy-γ-PyPyPy-Dp named MGB(3+3) (where γ is γ-aminobutyric acid, Py – *N*-methylpyrrole carboxamide and Dp – *N*,*N*-dimethylaminopropylamine residues) was synthesized in the laboratory by solid-phase method [36–37] and used as a polyamide component.

**Figure 5:**
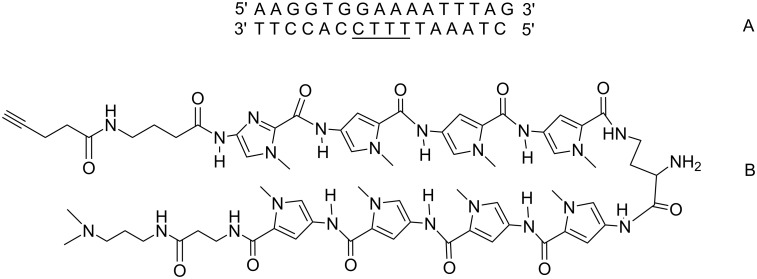
A) Sequence derived from the murine pericentromere repeat fragment with only one target site for the polyamide F1-NH_2_ [2–3]. The target sequence is underlined. B) Chemical formula of the polyamide F1-NH_2_.

The first model is interesting for an anti-HIV strategy, but it is not suitable for visualization of dsDNA because of the low concentration of integrated viral DNA in the genome. That is why for the synthesis of the fluorescent probes by "click chemistry" we decided to use a polyamide F1-NH_2_ that targets the tandem repeats of murine pericentromeres abundant in the murine genome [2–3]. The polyamide structure and its target dsDNA sequence fragment used in our previous work are shown on Figure 5.

In addition to the terminal alkyne group, an amine group was introduced into the α-position of the γ-aminobutyric acid linker (Figure 5B). This provides versatility for labelling of MGBs. For example, the terminal alkyne can be used for labelling of MGB with a fluorophore using "click chemistry" and the amino group can be used to conjugate the probe to TFO or to another polyamide [15] and vice versa.

### Synthesis of modified polyamides, containing an azide or alkyne group

**Figure 6:**
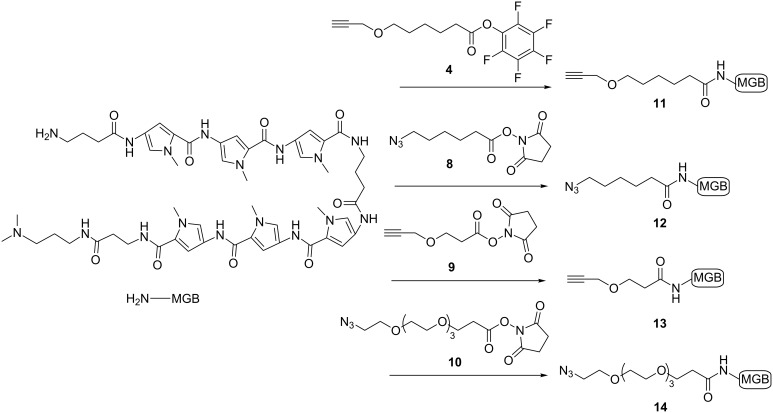
Synthesis of azide- and alkyne-modified MGBs.

The following polyamides (**11**–**14**, Figure 6) bearing either alkyne or azide groups were obtained via acylation of their *N*-terminal amine by the activated esters method in DMF in the presence of Hünig's base (see Supporting Information File 1 for experimental details). We have pursued several goals: 1) to synthesize a variety of modified MGBs with variable lengths of linkers in order to adapt conjugate structures for optimal interaction with their target sequences and eventually for DNA-templated coupling; 2) to synthesize both versions (azide and alkyne) of MGBs and TFOs. In addition, alkyne modified polyamides are suitable for an easy synthesis of fluorescent probes via their labeling by commercial and "home-made" fluorescent dyes bearing azide groups.

It was noted that yields of alkyne-modified MGBs were higher (50–83%) compared to those of azide modified MGBs (≈40%). The other variant for insertion of terminal azide or alkyne groups into the polyamides is a direct solid-phase synthesis using terminal azide or alkyne-containing carboxyl synthons as the last step of the MGB’s synthesis (see reference [2]).

### Synthesis and fluorescent properties of fluorescent probes based on polyamides

**Figure 7:**
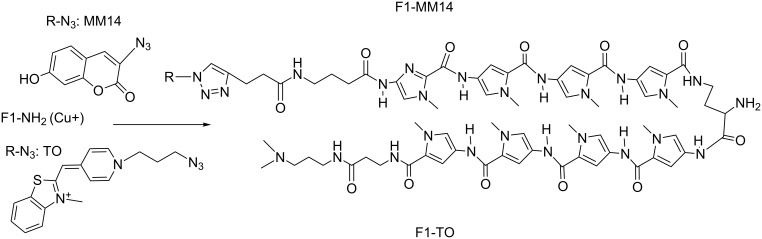
Structures of fluorescent probes synthesized by "click chemistry".

For fluorescent labeling of polyamides we used a polyamide F1-NH_2_ and two fluorophores bearing an azide functional group: one cyanine TO (derivative of thiazole orange [18]), and one coumarine MM14 kindly provided by M. P. Teulade-Fichou (Figure 7). The conjugation was conducted in a microwave reactor as for the polyamide tandem synthesis [2]. The details of the synthesis protocol and characteristics are described in Supporting Information File 1, Table S1.

**Figure 8:**
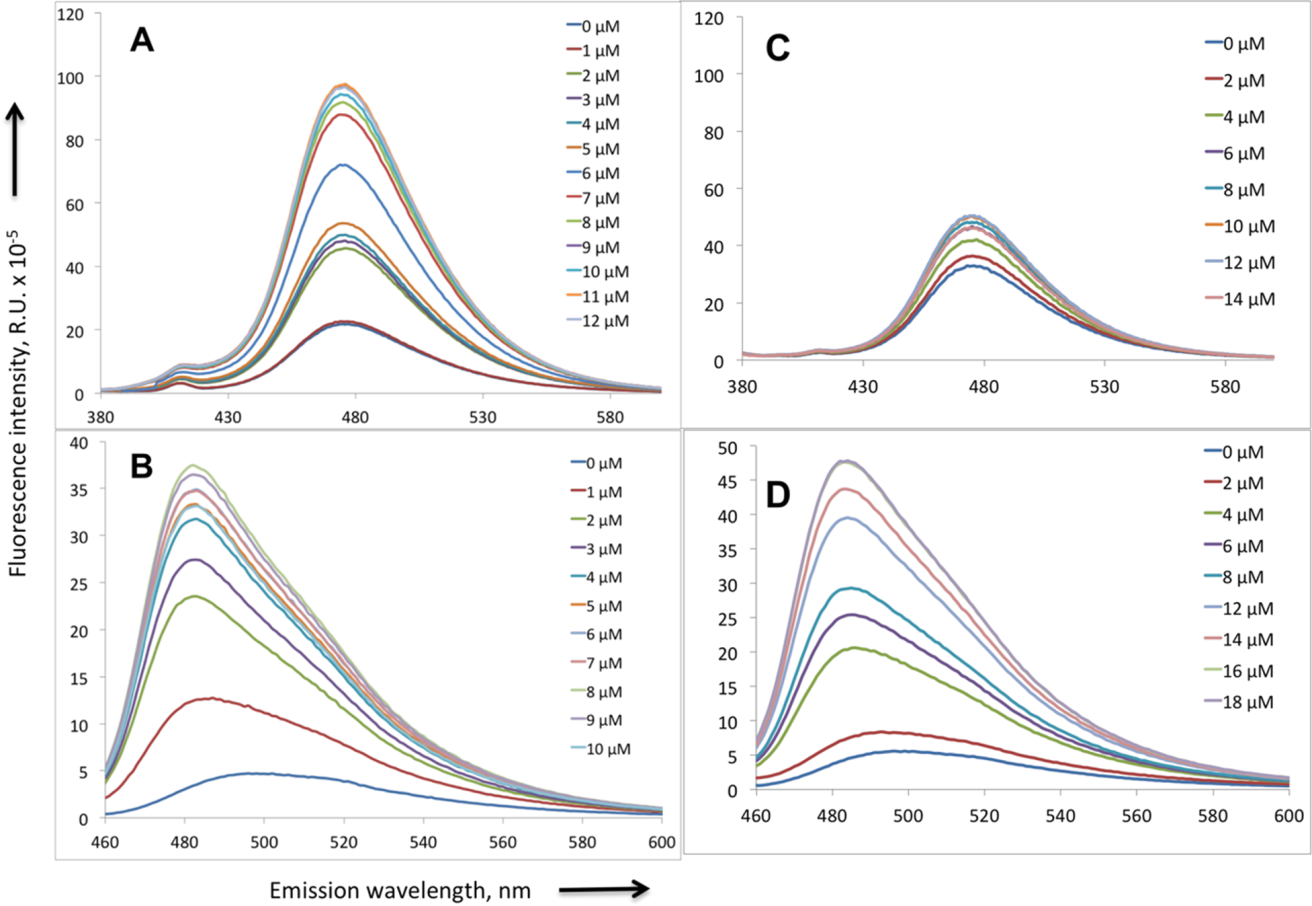
Titration of the probes F1-NH_2_-MM14 (12 µM, A, C) and F1-NH_2_-TO (10 µM, B, D) by the target DNA duplex (left) and non-cognate duplex (right) in 0.025 M tris-HCl, pH 7.5, at room temperature. The sequence of the target duplex is depicted in Figure 5A, the sequence of the non-cognate duplex is 5'-GGGTGAGGAGGAGGGAGATCGGTC-3'/3’-CCCACTCCTCCTCCCTCTAGCCAGT-5'. DNA concentration after each addition is indicated on the right panel of each picture.

Fluorescence properties of the probes were studied in the experiments on titration of the probes by the target DNA duplex (presented on Figure 5A). As it is seen in Figure 8, the fluorescence of the probes increases upon titration and is saturated at equimolar concentration of the target DNA. The total increase of the fluorescence intensity reaches 4.3 times for the F1-NH_2_-MM14 probe and 8 times for the F1-NH_2_-TO probe. In addition, a blue shift of the emission maximum of about 15–16 nm is observed upon interaction of the F1-NH_2_-TO probe with the target duplex. To test the specificity of these probes we titrated them with a non-target duplex that resulted in a 1.5 times increase in fluorescence emission for the MM14 probe and also 8 times increase for the F1-NH_2_-TO probe (Figure 8). The non-specific increase of the probe fluorescence has been observed in our previous work [2–3]. Thus, only F1-NH_2_-MM14 is sequence-specific. We ascribe this low specificity of F1-NH_2_-TO to strong intercalation properties of the TO fluorophore that can intercalate into DNA independently of the polyamide.

UV-visible absorption spectra in the interval 300–700 nm do not change upon DNA titration (data not shown), which means that increase in fluorescence emission is due to the increase of the quantum yield of fluorescence. Studies of interaction of the probes with fixed and live mouse cells are under investigation in the Paris laboratory.

### Synthesis of modified oligonucleotides containing an alkyne group from commercially available oligonucleotides

**Figure 9:**
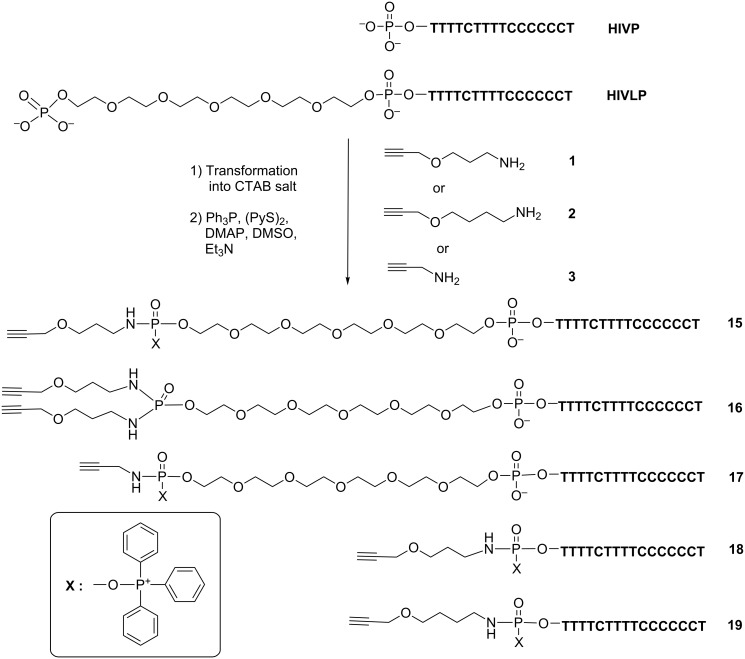
Synthesis of modified oligonucleotides containing an alkyne group.

A series of parallel TFOs, bearing different alkyne linkers at the terminal phosphate position (**15–19**, Figure 9) was prepared from commercially available phosphorylated oligonucleotides by activation of the terminal phosphate in oligonucleotides [11,22]. Two TFOs were purchased; one of them contained a long hexa(ethyleneglycol) linker that permits to connect two components of TFO-MGB conjugates located in major and minor grooves of dsDNA. In our previous work this linker permitted to obtain the most stable complexes of target DNA with TFO-MGB conjugates [11].

**Figure 10:**
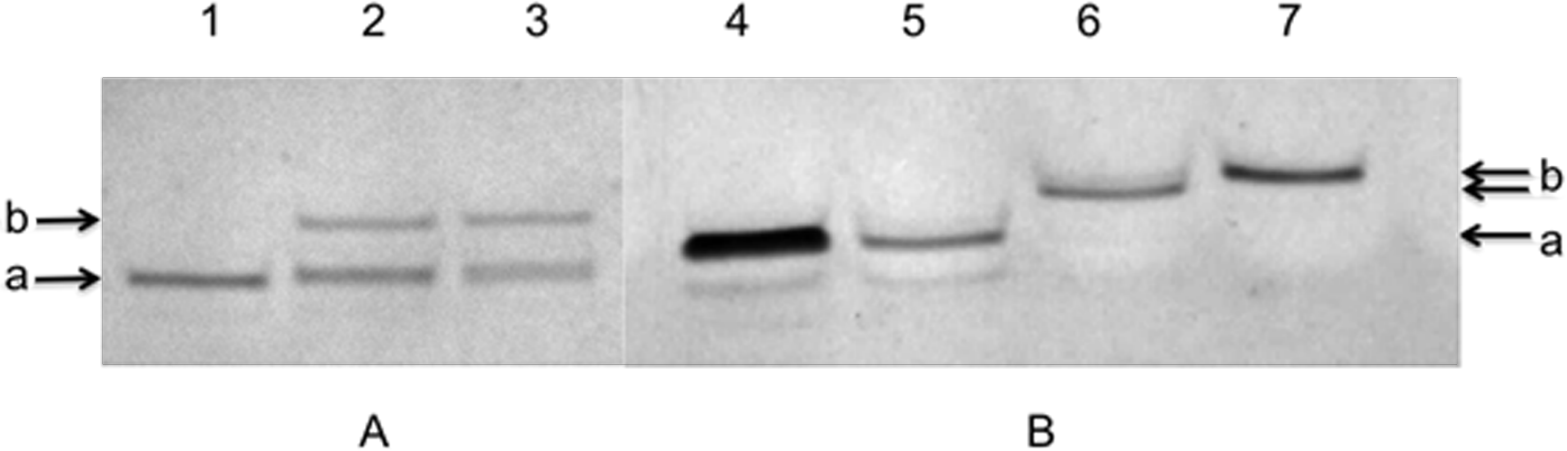
Gel electrophoresis of oligonucleotides modified by alkyne linkers: A – oligonucleotide HIVP (detection by UV-shadowing). Line 1 – control, starting oligonucleotide, lines 2 and 3 – reaction mixtures after its interaction with linker **1** (product **18**) and linker **2** (product **19**), respectively. B – oligonucleotide HIVLP modified by linker **1**. Line 4 – control, starting oligonucleotide, lines 5–7 – electrophoresis of individual products obtained after purification of reaction mixture by HPLC. 5 – initial oligonucleotide, 6 – first peak (product **15**), 7 – second peak (product **16**).

The resulting products were analyzed by 20% denaturing gel electrophoresis (PAGE) as described in [11]. For oligonucleotide HIVP products, we observed the formation of one slower migrating band on the PAGE gel with various yields of reactions (several examples are shown in Figure 10A). For oligonucleotides with a long linker HIVLP, except of the starting oligonucleotide, two barely resolved bands of products were observed. After purification by reversed-phase HPLC, three major peaks have been obtained; the first one was identified as a starting oligonucleotide, the second one – as a product **15** with only one linker **1** and the third one – as a product **16** with two linkers **1** connected to the same terminal phosphate of the hexaethylene phosphate moiety. This distribution of products has been previously observed [11,23].

Mass spectrometry identification of the resulting product gave quite surprising results. The molecular masses of mono-alkyne-modified oligonucleotides showed an additional mass equal to 260–262 Da. The molecular mass of the bis-modified HIVLP **16** corresponded well to the calculated one (Table S2 in Supporting Information File 1). This phenomenon has been already observed [24], though with quite low yields of products. These DNA derivatives were identified as products of incomplete departure of the oxidized triphenylphosphine moiety with the formation of the residue **X** covalently attached to the phosphorus of the terminal phosphate (Figure 9, insert). Attempts to eliminate this residue by treatment with aqueous 25% ammonia have not been successful. However, it was shown later that this residue does not prevent the subsequent CuAAC reactions with the azido-containing molecules.

In view of these results, we decided to synthesize TFOs directly by solid-phase synthesis using a 3'-terminal synthon possessing an alkyne group [30] in order to avoid additional post-synthetic reactions and formation of side products. Also we decided to profit from pH-independent triplex formation by antiparallel TINA-TFOs [17] and from fluorescent properties of TINA, which facilitate the electrophoretic analysis of reaction mixtures. Thus, we inserted two TINA moieties into antiparallel TFO according to our previous results.

**Figure 11:**
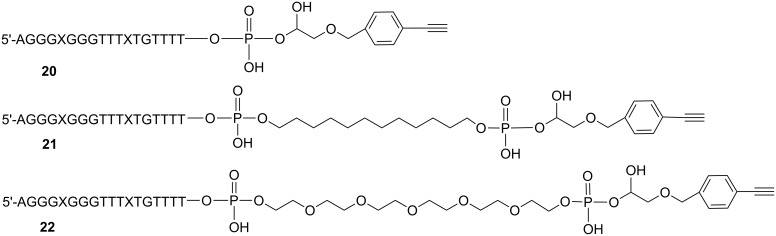
TINA-TFOs bearing a 3'-alkyne group for antiparallel triplex formation with the target HIV proviral DNA (Figure 4).

Three TINA-containing TFOs **20–22** possessing 3'-terminal alkyne groups (Figure 11) that differ in the length and nature of the 3'-linker were synthesized and used for further conjugations [17,30].

### Synthesis of polyamide-TFO conjugates by CuAAC reaction

**Figure 12:**
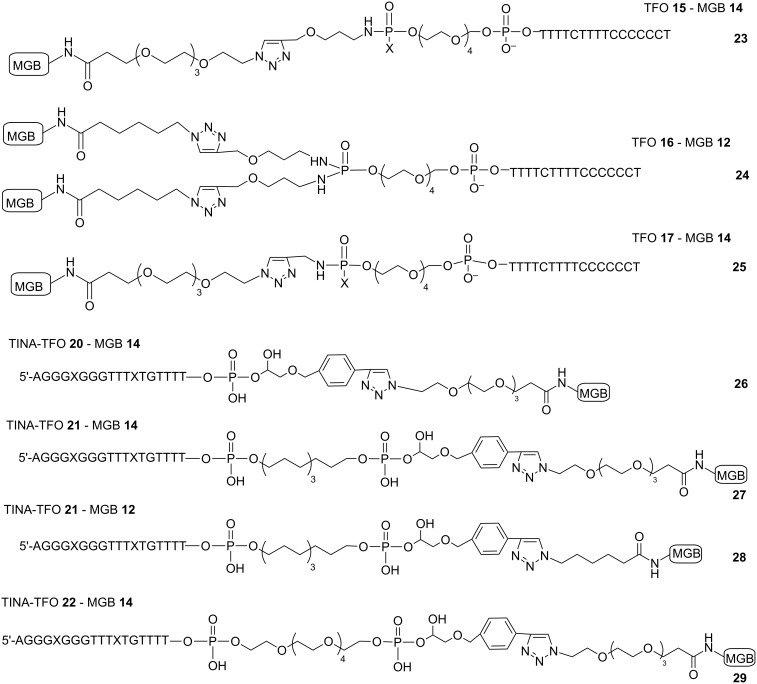
Structures of polyamide-TFO conjugates.

To establish conditions for the synthesis of TFO-MGB conjugates we used 5'-alkyne modified parallel TFOs (**15–17**) in combination with the *N*-terminal azide-modified MGBs **12** and **14** (see Figure 12, Figure 2B for the synthesis and Supporting Information File 1 for experimental details).

**Figure 13:**
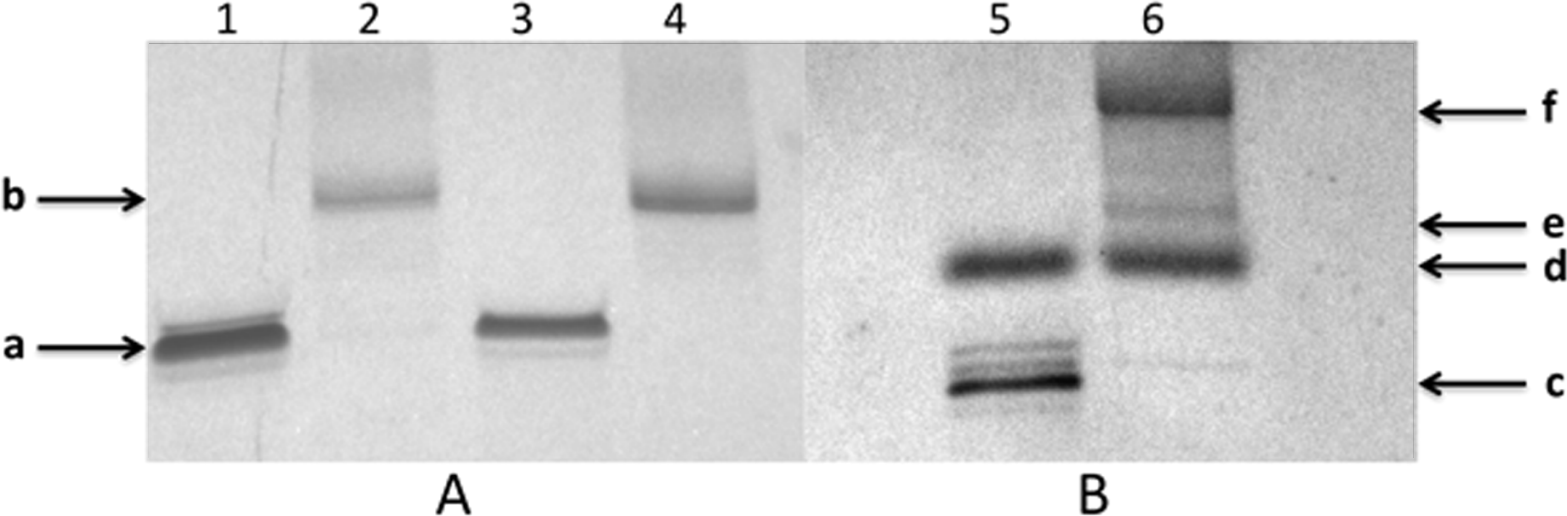
Electrophoresis analysis of samples from reaction mixtures after click reactions between alkyne-TFO and azide-polyamide in denaturing 20% polyacrylamide gel, visualization by UV-shadowing. A) conjugates **23** and **25**: lane 1 – control oligonucleotide **15**, lane 2 – conjugate **23**, lane 3 – control oligonucleotide **17**, lane 4 – conjugate **25**. B, conjugate **24**: lane 5 is control oligonucleotide **16**, lane 6 – conjugate **24**. The arrows indicate positions: a and c – initial alkyne-modified oligonucleotides, b and e – 1:1 conjugates TFO:polyamide, f – 1:2 conjugate TFO-polyamide, d – visible migration marker xylene cyanol.

Three combinations of the components were tried: TFO **15** and MGB **14** resulting in conjugate **23**, TFO **16** (bifunctional) + two MGBs **12** (conjugate **24**) and TFO **17** + MGB **14** (conjugate **25**). The “click” reaction proceeded smoothly with complete conversion of the starting TFO at room temperature and the formation of only one product (see 20% denaturing PAGE, Figure 13). In case of bifunctional TFO **16** the bis-polyamide product is mainly formed (Figure 13B, line 6, f) with barely visible traces of mono-polyamide conjugate (line 6, e). Lines 5d and 6d are visible by UV-shadowing bands of electrophoresis migration marker xylene cyanol that is absent in other gels.

**Figure 14:**
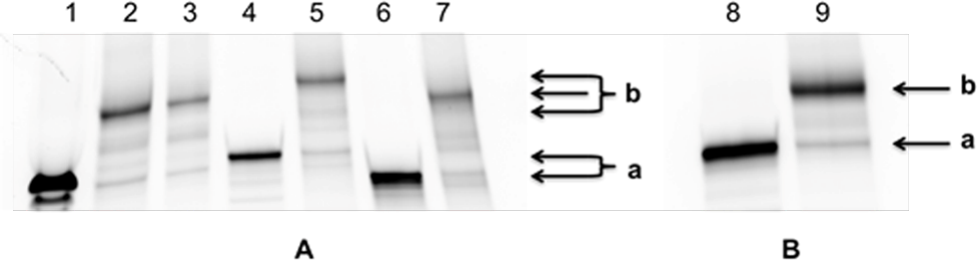
Electrophoresis analysis of reaction mixtures in 20% denaturing polyacrylamide gel after TINA-TFO-MGB conjugation (visualization by TINA fluorescence). A) Conjugates **26** (lanes 2 and 3), **27** (lane 5), **29** (lane 7). Lanes 1, 4 and 6 are control oligonucleotides **20**, **21** and **22**, respectively B) Conjugate **28** (lane 9); lane 8 – control oligonucleotide **21**. Arrows indicate: a, control oligonucleotides, b, conjugates.

Mass-spectrometry analysis confirmed the identity of the products obtained (Table S3 in Supporting Information File 1). Inspired by these results, we applied the same conjugation procedure to TINA-containing TFOs **20**–**22**. [17] Conjugation of these TFOs with MGB **12** and **14** (**26**: **20** + **14**; **27**: **21** + **14**; **28**: **21** + **12** and **29**: **22** + **14**) proceeded with quite good yields and purity of the products (Figure 14).

Purification of TINA oligonucleotides by reversed phase HPLC was quite difficult. Due to the high hydrophobicity of the pyrene moiety the retention time of the parent oligonucleotide and the conjugate were very close to each other and their peaks were overlapped. Thus preparative denaturing gel electrophoresis was used for their final purification.

### DNA-templated synthesis of TFO-MGB conjugates

It has been shown that the presence of copper ions does not prevent duplex and triplex formation and the DNA-directed cycloaddition reaction proceed smoothly on these templates [38]. In 2003, the team of P. Dervan succeeded to obtain a conjugate of two polyamides by template-directed synthesis on the adjacent sequences of a target DNA fragment using the Huisgen 1,3-cycloaddition reaction even without copper catalysis [39]. The rational for this type of synthesis was to find an optimal orientation of two DNA-binding ligands for the synthesis of highly affine conjugates and to provide a favorable approaching and orientation of the two components in order to facilitate the reaction. However, after this publication the use of a Huisgen reaction without copper catalysis in the template-directed synthesis was never reproduced.

In the present work, we tried to approach and correctly orient two components (TFO and polyamide) by their binding on the target dsDNA sequence as a template. Linkers of different lengths and configurations were used in order to select one that fits better the DNA architecture.

**Figure 15:**
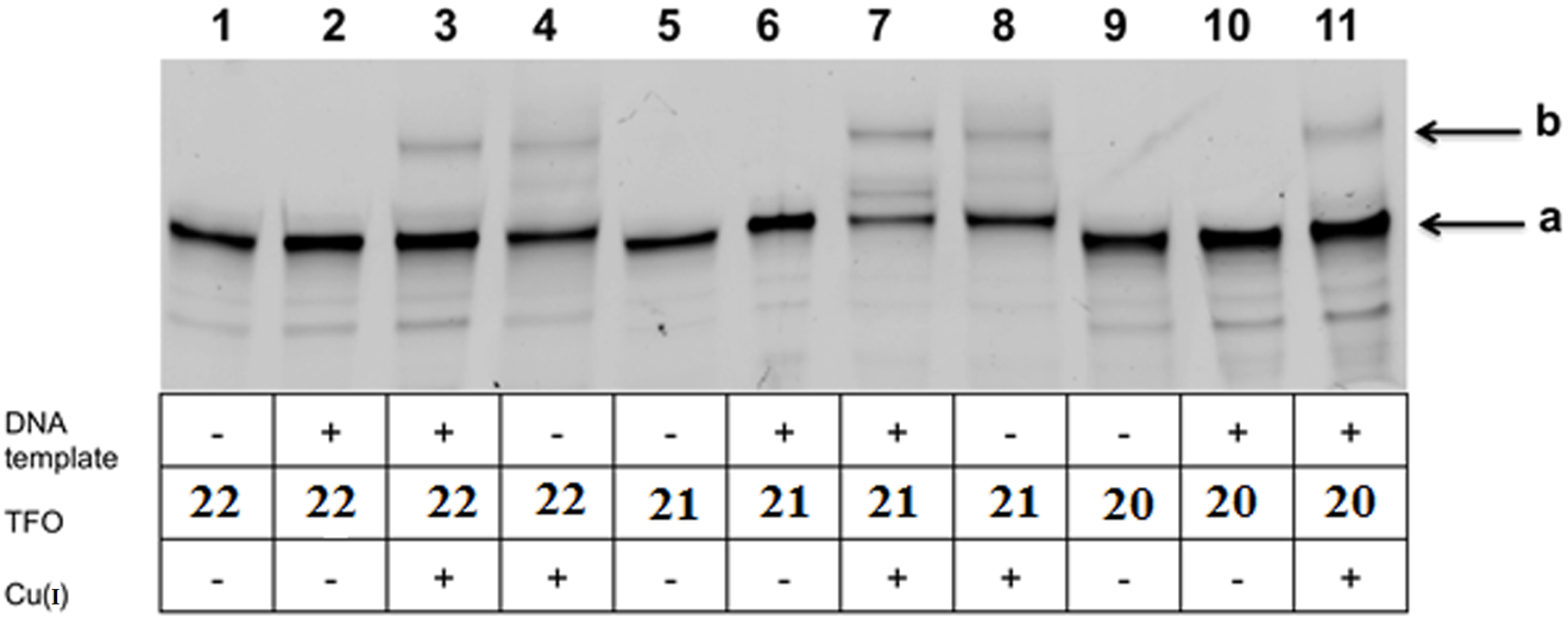
Electrophoretic analysis of reaction mixtures in standard 20% denaturing PAGE after DNA-templated synthesis. All reaction mixtures in the 0.1 M TEA-acetate buffer, pH 7.0 contained 10 µM polyamide **14** and components, indicated in the table. The concentrations of the DNA template (target duplex shown in Figure 3) and TINA-TFOs (shown in Figure 10) were 10 µM. Cu(I) was added as a mixture of components up to the final concentrations of CuSO_4_ and THPTA 5 mM and sodium ascorbate 10 mM.

Only TINA-TFOs that form stable triplexes at neutral pH and relatively high temperatures [40–41] are suitable for these trials. Oligonucleotides **20**–**22** and azido-modified polyamide **14** (soluble in water) were chosen for DNA-template experiments. Reactions were performed with equal concentrations (10 µM) of TINA-TFO, polyamide and the target duplex (Figure 4), first at room temperature during 6–24 h and then at 37 °C for six or more hours (see Supporting Information File 1). Experiments were carried out with each TINA-TFO in the conditions used for triplex formation in four variants: 1) TFO + polyamide, no Cu(I) and template, 2) Template duplex + TFO + polyamide without copper catalysys, 3) TFO + MGB, no template, with copper catalysis, 4) Template duplex + TFO + polyamide with copper catalysis. Initially, a triplex was formed by mixing and incubation of the duplex with the TINA-TFO, then polyamide and Cu(I) with necessary reagents were added. Standard 20% denaturing PAGE analysis (Figure 15) demonstrated that the conjugation occurs only in the presence of copper ions, however, with quite low yields, possibly due to low concentration of components. The effect of the DNA template on the formation of conjugates was negligible.

### Interaction between MGB-TFO conjugates and the target duplex

**Figure 16:**
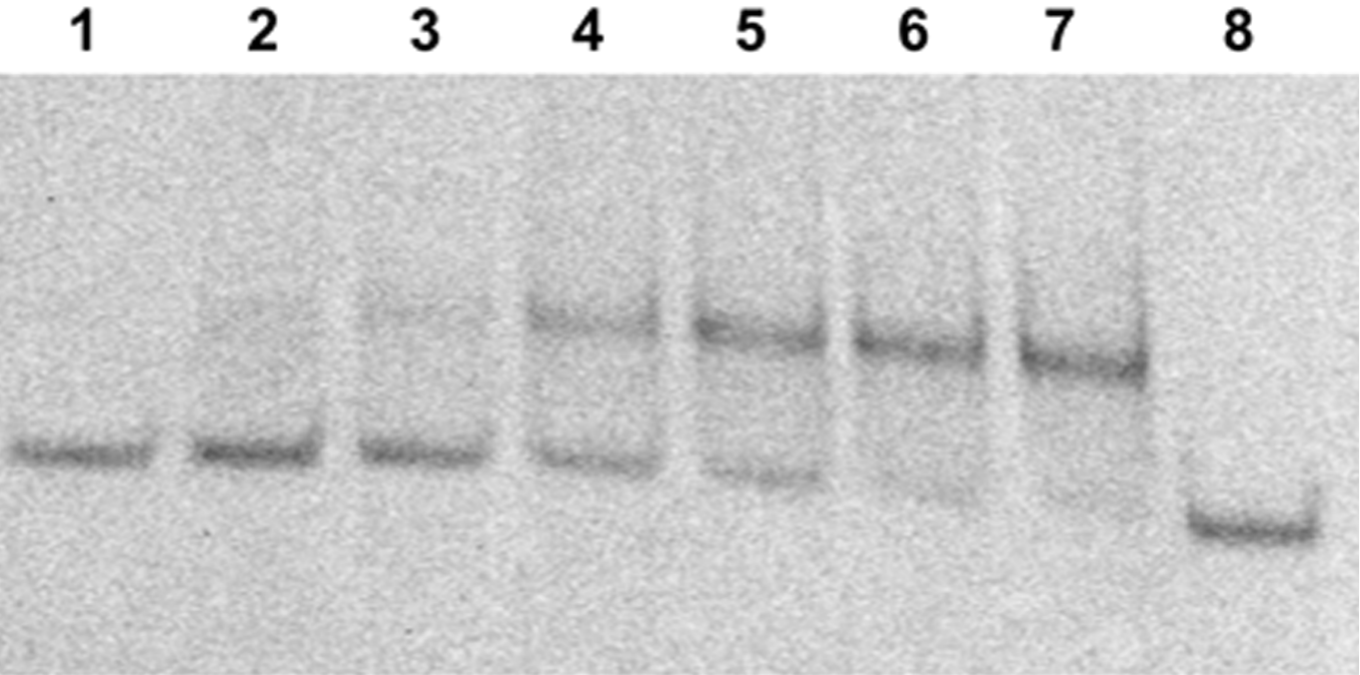
Non-denaturing gel electrophoresis analysis of conjugate **28** with fluorescein-labeled target HIV duplex (concentration 120 nM), polyamide concentrations: 0, 0.45, 0.75, 1.13, 1.5, 2.25, 3, 0 μM (lines 1 to 8, respectively) in 0.05 M HEPES, 50 mM NaCl, 5 mM MgCl_2_, pH 7.2. Visualization of the gel was performed by monitoring fluorescence of the fluorescein-labeled duplex depicted in Figure 4.

Our previous studies with parallel TFO-polyamide conjugates demonstrated that conjugated polyamides poorly stabilized both DNA and 2'-*O*-methyl-RNA parallel triplexes, and there were independent interactions between polyamide or TFO component and the target duplex. Sufficient stabilization of triple complexes at neutral pH occurs only in case of TFO-bis-polyamide conjugates in the form of antiparallel tandem [11,14,34]. No stabilization of complexes between mono-conjugates (TFO:1 polyamide) and target duplexes at neutral pH (7.2–7.5) was observed in gel-shift and thermal denaturation experiments [11]. The same results were obtained with conjugates **15**–**19** (data not shown). It was more interesting to study the interaction between conjugates of TINA-modified TFOs and target duplexes. Thermal denaturation studies are not suitable for experiments with TINA oligonucleotides because the melting of the triplex is not visible on their melting curves due to the low hyperchromic effect of G-C:G triplets and the high stability of antiparallel triplexes that dissociate together with target duplexes [17]. We used gel-shift experiments in order to measure apparent dissociation constants between conjugates and target duplexes [42]. A gel mobility assay has been performed in neutral buffer (pH 7.2) and at room temperature in order to approach the physiological conditions. For antiparallel TINA-TFO-MGB conjugates **26**–**29**, the triplex formation has been observed by the gel-shift method both for initial TINA-TFOs and for TINA-TFO-polyamide conjugates, but no improvement on affinity was detected. The best results were obtained for the conjugate **28.** The gel image is shown on Figure 16.

The dissociation constants were calculated after quantification of the gel by graphical method [42]. In the case of conjugate **28**, this constant was 1.15 ± 0.01 µM that is even higher than that for the triplex formed by the parent TINA-TFO without any linkers attached (0.16 ± 0.02 µM), parent TINA-TFO with attached 3'-hexaethyleneglycol phosphate linker (EG)6p (0.45 ± 0.06 µM) and the conjugate of the latter with polyamide ([TFO]-(EG)6p-γ-MGB) obtained via Mukaiyama reaction, as in [11] (0.30 ± 0.03 µM) [43]. Other three conjugates (**26**, **27** and **29**) demonstrated even lower affinity and appearance of the smear between bands in the gel (data not shown). These results indicate that the presence of the triazole moiety in the linker slightly perturbs the triplex formation.

## Conclusion

Diverse bifunctional linkers of different length, hydrophobicity and flexibility for insertion of terminal azide or alkyne groups into biomolecules were synthesized and applied for oligonucleotides and polyamide MGBs. A simple and effective method of bioorthogonal conjugation using CuAAC was applied to the synthesis of conjugates between various TFOs and MGBs. Synthesized products were purified, isolated and characterized. Functionalized polyamides were applied for the synthesis of fluorescently labeled probes for detection of native dsDNA using fluorophores bearing azide groups.

Attempts to apply template-directed coupling between TINA-modified TFOs and polyamides were unsuccessful. No effect of the DNA template was observed on the yield of oligonucleotide-polyamide conjugates. Copper(I) catalysis is absolutely necessary for a successful Huisgen 1,3-cycloaddition reaction.

Physicochemical studies of the interaction between the obtained conjugates and target dsDNA confirmed our previous results with the conjugates of DNA and 2'-*O*-methyl-RNA that one molecule of the polyamide attached to oligonucleotide does not improve the DNA triplex formation. Even in the case of TINA-TFOs with improved triplex-forming properties at physiological pH, there was no improvement of the probe affinity. This can be explained by a destabilizing effect of the linker-polyamide construction on the DNA-conjugate interaction or the non-optimal structure of linkers that connect two components. Additional studies aiming to optimize the structure of the linker using molecular modeling are necessary.

## Supporting Information

Synthesis and characteristics of the linkers; conditions of azide- and alkyne-modified MGBs synthesis and mass spectral characteristics of the obtained products (**11**–**14**); synthesis of fluorescent probes based on polyamides and their characteristics (Table S1); synthesis of modified oligonucleotides containing alkyne group (**15**–**19**) and mass spectrometry identification (Table S2); details of synthesis of Polyamide-TFO conjugates by CuAAC reaction and mass-spectrometry characteristics (Tables S3 and S4, Figure S1) are provided in Supporting Information File 1.

File 1 Experimenal and analytical data.
