# Supplementary material for: Application of Cu(I)-catalyzed azide–alkyne cycloaddition for the design and synthesis of sequence specific probes targeting double-stranded DNA
Source: Beilstein J Org Chem. 2016 Jun 30;12:1348–60. doi: 10.3762/bjoc.12.128 (PMC4979877; doi:10.3762/bjoc.12.128)
Supplement: File 1 — Experimenal and analytical data. [file Beilstein_J_Org_Chem-12-1348-s001.pdf]

Supporting Information

for

Application of Cu(I)-catalyzed azide–alkyne cycloaddition for the design and synthesis of sequence specific probes targeting double-stranded DNA

Svetlana V. Vasilyeva[1]*, Vyacheslav V. Filichev[2] and Alexandre S. Boutorine[3]

Address: [1]Institute of Chemical Biology & Fundamental Medicine, SB of RAS, pr. Lavrent'eva 8, 630090 Novosibirsk, Russia and [2]Institute of Fundamental Sciences, Massey University, Private Bag 11-222, 4442 Palmerston North, New Zealand and [3]Structure and Instability of Genomes, Sorbonne Universités, Muséum National d'Histoire Naturelle, INSERM U 1154, CNRS UMR 7196, 57 rue Cuvier, C.P. 26, 75231 Paris cedex 05, France

Email: Svetlana Vasilyeva - svetlana2001@gmail.com

*Corresponding author

Experimental

General information

Reagents and solvents were purchased from Sigma-Aldrich (USA), Fluka (Switzerland), Merck, Acros Organics (USA), Carlo Erba and used without further

purification. Anhydrous solvents were either purchased or obtained by distillation and stored above dried molecular sieves. Non-modified oligonucleotides were purchased from Eurogentec (Seraing, Belgium). Reactions of polyamide modification were monitored by TLC carried out on 0.25 mm Merck silica-gel plates (60F-254). Merck silica gel (60, particle size 0.040–0.063 mm) was used for flash-column chromatography. Tris(hydroxypropyl)triazolmethylamine (THPTA) was kindly provided by Prof. T. Brown (Department of Chemistry, University of Oxford, UK), coumarin fluorophore MM14 – by M. P. Teulade-Fichou (Institute Curie, Orsay, France). Monomers for polyamide synthesis were purchased from Bachem (Germany) and PolyPeptide Laboratories (France). Activating reagent HATU (2-(1H-7-azabenzotriazol-1-yl)-1,1,3,3-tetramethyluronium hexafluorophosphate) and HOAT (1-hydroxy-7-azabenzotriazole) were from 'Molekula' (UK). Synthesis of *N*-methylpyrrole/*N*-methylimidazole polyamides was carried out using manual solid-phase peptide synthesis on Boc-ß-ala-PAM resin from Merck (>0.5 mmol/g, 100–200 mg) at room temperature in a 1 × 12 cm column with a glass filter [1,2]. The coupling of the terminal alkyne or azide group was realized as in [3] using appropriate synthons.

Instrumentation

Reversed-phase HPLC analysis and purification were performed on Agilent Technologies 1200 analytical and preparative chromatographs piloted by the Agilent ChemStation program. Mass-spectrometry analysis by electrospray Q-TOF (ESI Q-TOF MS) was performed on a Q-Star instrument (Applied Biosystems, Courtaboeuf, France) in the positive (for polyamides) or negative (for oligonucleotides) mode; analyses were performed in methanol or water, respectively. Polyacrylamide gels with fluorescently labeled oligonucleotides and conjugates were scanned on a

Typhoon 9410 (Amersham) fluorescence scanner with laser excitation at 532 nm and emission filter at 526 nm. Non-labeled oligonucleotides and their conjugates were detected after polyacrylamide gel-electrophoresis by "UV-shadowing" method on fluorescent screen in SynGene G-box gel imaging system. UV–vis spectra and thermal denaturation curves were obtained on a UV-visible spectrophotometer "Uvicon XL" (Secomam, Nova Analytic Company) with 12 quartz cells installed in a mobile thermostated support piloted by ThermAlys software (DuDoTec GmbH). Fluorescent TINA oligonucleotides were visualized in GBox by fluorescence on Transilluminator with excitation wavelength 302 nm. NMR spectra were recorded on Bruker AVANCE 400 NMR spectrometer equipped with a ^{1}H broadband reverse gradient probe head.

Synthesis of carboxyl linkers, containing azide group

Synthesis of 3-propynyloxypropylamine linker **1** was carried out as described in [4], 4-propynyloxybutylamine **2** – as in [5], 6-propynyloxyhexanioc acid pentafluorophenyl ester **4** – as in [6] and *N*-hydroxysuccinimide ester of 6-azidohexanoic acid **8** – as in [7]. A general procedure for the synthesis of linkers with different lengths bearing an azide group (5-azidovaleric acid (**5**), 6-azidohexanoic acid (**6**) and 11-azidoundecanoic acid (**7**)) has been realized according to the method described in [7] from the corresponding bromo-substituted acids. Yields of **5**, **6**, and **7** were 74%, 78% and 80% respectively. Spectral characteristics of the resulting products **5** and **6** correspond to the previously described [4,8]. Spectral characteristics of the **7**: ^{1}H NMR ($CDCl_3$, 400 MHz): δ ($CHCl_3$ = 7.26 ppm), 3.27 (t, 2H, CH_2N_3), 2.37 (t, 2H, CH_2COOH), 1.65 (m, 4H, $CH_2CH_2(CH_2)_6CH_2CH_2$), 1.43 (m, 12H, $CH_2CH_2(CH_2)_6CH_2CH_2$);. ^{13}C NMR (CDCl3, 100 MHz): δ ($CHCl_3$ = 77.33 ppm), 179.88 (C=O), 51.49 (CH_2N_3), 33.99 (CH_2COOH), 29.37 – 29.01

(($CH_2)_5CH_2CH_2COOH$), 28.8 ($CH_2CH_2N_3$), 26.4 ($CH_2CH_2CH_2N_3$), 24.6 (CH_2CH_2COOH). IR: N=N=N 2090 cm^{-1}. MS-ESI (m/z): $[M-H]^-$ calculated 143.07, found 143.14 for **5**; $[M-H]^-$ calculated 158.09, found 157.21 for **6**; $[M-H]^-$ calculated 227.16, found 227.33 for **7**.

A general procedure for post-synthetic terminal functionalization of polyamides by azide or alkyne moieties 11–14

The activated ester of appropriate acid **4**, **8**, **9** or **10** (94 µmol, 10 equiv) was added to polyamide (10 mg, 9.4 µmol, 1 equiv), dissolved in 200 µL of DMF and 80 µL of *N*-ethyldiisopropylamine (470 µmol, 50 equiv). After incubation for 2 h at room temperature, the reaction mixture was precipitated by ether. The analysis of the precipitate by RP-HPLC and TLC showed completion of the reaction. The product was purified by semi-preparative HPLC on a C-18 X-Terra (7µm) column (7,7 × 300 mm; Waters), using a linear gradient of 0–80% MeCN in H_2O containing 0.1% of TFA at a flow rate of 2 mL/min. After solvent removal, the product (**11–14**) was dried in vacuo. Yields were 50 %, for **11,** ES-Q-TOF-MS: 1228.24 ($[M-H]^-$; calc. 1228.63); 17% for **12**, ES-Q-TOF-MS: 1215.3 ([M-H]-; calc. 1215.62); 83 % for **13**, ES-Q-TOF-MS: 1186.17 ([M-H]-; calc. 1186.32); 39 % for **14,** ES-Q-TOF-MS: 1349.13 ([M-H]-; calc. 1349.68).

Coupling of the azide-bearing fluorophore group to the alkyne-modified polyamide

The synthesis of 2-{[1-(3-azidopropyl)pyridin-4(1*H*)-ylidene]methyl}-3-methylbenzo[d]thiazol-3-ium (**TO,** thiazole orange) has been described in [9]. Conjugation of **TO** and the coumarin fluorophore (3-azido-7-hydroxy-2*H*-chromen-2-one, **MM14)** to the alkyne-modified polyamide **F1-NH2** was realized in a microwave

reactor Monowave 300 (Anton Paar) in a small reaction tube of total volume 4 mL. Equimolar solution of polyamide **F1-NH2** and fluorophore (1–2 µmol) was dissolved in 300 mL of DMSO. 70 µL of 50 mM aq $CuSO_4$/Tris[1-(hydroxypropyl-1,2,3-triazol-4-yl)methyl]amine (THPTA), 70 µL of 0.1 M aq sodium ascorbate and 70 µL of 1 M tris-TEA buffer, pH 7.0, were added and the volume of the reaction mixture was adjusted by water to 700 µL. The mixture was irradiated in a microwave reactor for 20 min at 90 °C, treated by chelating resin Chelex-100 from BioRad (3 equiv of chelating groups relative to copper ions concentration) and the product was purified by RP-HPLC using the same conditions as for the synthesis of the parent polyamides and characterized by UV–vis spectra and mass-spectrometry (see Table S1).

Table S1: Yields and properties of fluorescent probes obtained by CuACC.

Probe	Yield, %	M.W. calculated	M.W. obtained	λ_{ex}, nm	λ_{em}, nm
F1-TO	58	1739.80	1738.80	440	480-495
F1-MM14	62	1618.73	1618.70	360	475

Synthesis of TFOs 15–19 bearing terminal alkynes

Modified oligonucleotides, bearing one or two alkyne linkers at the 5’-end, were prepared by Mukaiyama reaction [10] according to protocols described for oligonucleotide conjugates [11]. 4-(Dimethylamino)pyridine (DMAP; 5 mg, 40 µmol), dipyridyl disulfide (13,2 mg, 60 µmol), and Ph_3P (15,7 mg, 60 µmol), each dissolved in 50 µL of anhydrous DMSO, were added to a DMSO solution of the oligonucleotide in the form of cetyltrimethylammonium salt (120–200 µg, 40–65 nmol) dissolved in anhydrous DMSO (100 µL). The mixture was shaken for 20 min. Then, a solution of the alkyne linker (3-propynyloxypropylamine **1** or 4-propynyloxybutylamine **2** or

propargylamine **3**, 1–5 mg) was added, and the mixture was incubated at rt for 2 h, followed by precipitation with 3% $LiClO_4$ solution in acetone (1 mL). The precipitate was centrifuged to form a pellet that was washed with acetone, dried and dissolved in H_2O. The products **15–19** were purified by HPLC on a C-18 X-Terra (7 µm) column (7,7 × 300 mm; Waters), using a linear gradient of 5-50% MeCN in 0.02M aq NH_4OAc solution, pH 6.0, at a flow rate of 2 mL/min. Then solvent was removed in vacuo. The yields were 20–38% for the mono-modified oligonucleotide and 24% for the bis-modified HIVLP-oligonucleotide. The purities of modified oligonucleotides were confirmed by 20% denaturing PAGE (7 M urea). Mass spectra and yields of obtained alkyne-modified oligonucleotides **15–19** are shown in Table S2.

Table S2: Mass spectra and yields data of obtained alkyne-modified oligonucleotides.

№	Designation	Yield after HPLC purification, %	M.W. calculated	M.W. obtained
**15**	HIVLP-L**1**	37	5577.68 [a]	5577.10
**16**	HIVLP-(L**1**)$_2$	24	5412.83	5412.20
**17**	HIVLP- L**3**	21	5519.60 [a]	5519.29
**18**	HIVP-L**1**	20	5233,39 [a]	5232,90
**19**	HIVP-L**2**	26	4987.42	4986,83

[a]Taking into account the residue X.

Synthesis of polyamide-TFO conjugates (23-29) by CuAAC reaction

A solution of modified oligonucleotide, bearing one or two alkyne linkers (170–350 µg, 30–55 nmol) in H_2O (35–40 µL) was mixed with a solution of azido-modified MGB

(10 equiv) in DMSO (40 µL). Then, a stock aq solution of 50 mM $CuSO_4$/THPTA (20 equiv) and 0.1 volume of 1 M triethylammonium acetate buffer pH 7 were added. The mixture was purged by argon for 30 s; 0.1 M aq sodium ascorbate (20 equiv) was added last. The reaction mixture was left overnight at room temperature, and then the mixture was precipitated with 3% $LiClO_4$ solution in acetone. The precipitate was centrifuged to form a pellet that was washed with acetone, dried, and dissolved in H_2O. The solution was treated for 30 min by chelating resin Chelex-100 from BioRad (3 equiv of chelating groups relative to the concentration of copper ions) that was then removed by filtration or centrifugation at 10,000 rpm in Eppendorf microcentrifuge. The products **23–29** were purified by HPLC on a C-18 X-Terra (7 µm) column (7.7 × 300 mm; Waters), using a linear gradient of 5–100% MeCN in 0.02M aq NH_4OAc at a flow rate of 2 mL/min. Then solvent was removed in vacuo. The yields were 30–90 % (see Tables S3 and S4).

Table S3: Conjugates of polyamides and parallel TFOs.

№	Components of the conjugates	Yield after HPLC purification, %	M.W. calculated	M.W. obtained
**23**	TFO **15** -MGB **14**	50	6929.50 [a]	6927.05
**24**	TFO **16 -**MGB **12**	32	7843.61	7843.05
**25**	TFO **17**- MGB **14**	90	6870.97 [a]	6873.09

[a]Taking into account the residue X.

Table S4: Conjugates of polyamides and antiparallel TINA-TFOs.

Number	Composition of the conjugate	Yield after HPLC purification, %

**26**	TINA-TFO **20** + polyamide**14**	29
**27**	TINA-TFO **21**+ polyamide **14**	41
**28**	TINA-TFO **21**+ polyamide**12**	71
**29**	TINA-TFO **22** +polyamide **14**	40

The purities of conjugates were confirmed by 20% denaturing PAGE (7 M urea). Purification of TINA oligonucleotides by reverse phase HPLC was quite difficult. Due to high hydrophobicity of pyrene moiety the retention time of the parent oligonucleotide and the conjugate were very close to each other and their peaks were overlapped. Thus preparative denaturing gel electrophoresis was used for their final purification (see below).

The most deceiving surprise was a rapid degradation of the HPLC-purified conjugates even after one-day storage in the freezer (Figure S1).

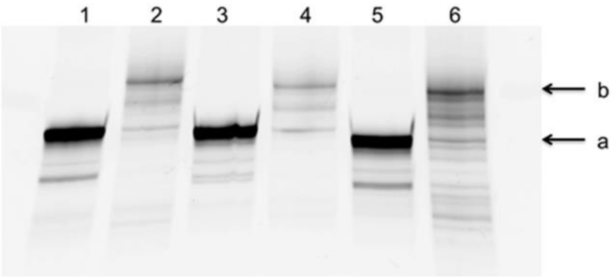

Figure S1: Conjugates of TINA_TFO and polyamides after purification by RP-HPLC and 1-day storage in the freezer at –20 °C. Lanes 2, 4, 6 – conjugates **26**, **27** and **29**, respectively, lanes 1, 3, 5 – control oligonucleotides **20**, **21**, **22**, respectively. Arrows indicate positions of: a, control oligonucleotides, b, conjugates.

The reason of this degradation of oligonucleotides is the DNA cleavage catalyzed by copper ions. They seem to be retained by conjugates after all the separation procedures and even after purification by HPLC (probably, as DNA

phosphate counterions). The solution of this problem was found in immediate filtration of the reaction mixture through the chelating resin Chelex 100 from BioRad. This resin allowed us to remove copper ions completely and to save the newly synthesized TINA-TFO-polyamide conjugates from decomposition.

As an alternative method of purification we also tried a preparative denaturing gel electrophoresis in 1 mm thick 20% polyacrylamide gel – tris-borate-EDTA – 7 M urea. Here, the good resolution of conjugates, initial products and degradation products has been observed. After excision of the product band and water extraction, a homogeneous pure conjugate was obtained. However, the yield of this procedure was quite low (about 20%).

Gel-shift experiments

Gel-shift experiments were performed in 15% non-denaturing polyacrylamide gels in 50 mM HEPES (pH 7.2), 50 mM NaCl and 5 mM $MgCl_2$. Each sample (10 µL) in the same buffer contained 1.2 pmol of fluorescein-labeled target HIV duplex with covalently linked complementary strands via four thymidine nucleotides (see publication [12]), glycerol (up to 8%) and eventually 0.06% bromophenol blue and xylene cyanol as migration markers. The mixture was first heated at 90 °C for 3 min and slowly cooled, then the third strand (conjugates **23–29**) was added up to its specific final concentration in each tube (0.45–3 µM). The mixtures were incubated at +4 °C overnight and then loaded onto 15% non-denaturing polyacrylamide gel. The electrophoresis was performed at a power 5 W for several hours. The gels were scanned on Typhoon using a laser at 473 nm and a 510LP filter to detect the fluorescein label. The image was analyzed using the ImageQuant or ImageJ

software. Apparent dissociation constants were calculated according to the protocol described in publication [12].

References

1. Krutzik, P. O.; Chamberlin, A. R. In *Combinatorial Library Methods and Protocols, Methods in Molecular Biology*; English, L.B. Ed.; Humana: Totowa, NJ, **2002**, Vol. 6. DOI: 10.1385/1-59259-285-6:77.
2. Krutzik, P. O.; Chamberlin, A. R. *Bioorg. Med. Chem. Lett.* **2002**, *12*, 2129-2132. DOI: 10.1016/S0960-894X(02)00359-1.
3. Nozeret, K.; Bonan, M.; Yarmoluk, S. M.; Novopashina, D. S.; Boutorine, A. S. *Bioorg. Med. Chem.* **2015**, *23*, 5932-5945. http://dx.doi.org/10.1016/j.bmc.2015.06.062.
4. Vasilyeva, S. V.; Budilkin, B. I.; Konevetz, D. A.; Silnikov, V. N. *Nucleosides, Nucleotides Nucleic Acids* **2011**, *30*, 753-767. DOI: 10.1080/15257770.2011.595379.
5. Kupryushkin, M. S.; Konevetz, D. A.; Vasilyeva, S. V.; Kuznetsova, A. S.; Stetsenko, D. A.; Pyshnyi, D. V. *Nucleosides, Nucleotides Nucleic Acids* **2013**, *32*, 306-319. DOI: 10.1080/15257770.2013.787147.
6. Vasilyeva, S. V.; Levina, A. S.; Li-Zhulanov, N. S.; Shatskaya, N. V.; Baiborodin, S. I.; Repkova, M. N.; Zarytova, V. F.; Mazurkova, N. A.; Silnikov, V. N. *Bioorg. Med. Chem.* **2015**, *23*, 2168-2175. http://dx.doi.org/10.1016/j.bmc.2015.02.063.
7. Vasilyeva, S. V.; Silnikov, V. N.; Shatskaya, N. V.; Levina, A. S.; Repkova, M. N.; Zarytova, V. F. *Bioorg. Med. Chem.* **2013**, *21*, 703-711. http://dx.doi.org/10.1016/j.bmc.2012.11.057.

8. McGeary, R. P. *Tetrahedron Lett.* **1998**, *39*, 3319-3322. http://dx.doi.org/10.1016/S0040-4039(98)00480-8.

9. Doluca, O.; Hale, T. K.; Edwards, P. J. B.; González, C.; Filichev, V. V. *ChemPlusChem* **2014**, *79*, 58-66. DOI: 10.1002/cplu.201300310.

10. Hashimoto, M.; Ueki, M.; Mukaiyama, T. *Chem. Lett.* **1976**, *5*, 157-160. DOI: 10.1246/cl.1976.157.

11. Grimm, G. N.; Boutorine, A. S.; Hélène, C. *Nucleosides, Nucleotides Nucleic Acids* **2000**, *19*, 1943-1965. DOI: 10.1080/15257770008045470.

12. Boutorine, A. S.; Escudé, C. in *Current Protocols in Nucleic Acid Chemistry* 7.12.1-7.12.16. Biophysical Analysis of Triple-Helix Formation, Jones, R.; Herdewijn, P., Eds.; John Wiley & Sons, Inc. **2007**, DOI: 10.1002/0471142700.nc0712s29.
